# Influence of Air Quality on Pro-environmental Behavior of Chinese Residents: From the Perspective of Spatial Distance

**DOI:** 10.3389/fpsyg.2020.566046

**Published:** 2020-09-15

**Authors:** Guanghua Sheng, Jiatong Dai, Hong Pan

**Affiliations:** ^1^Department of Marketing, School of Business, Jilin University, Changchun, China; ^2^School of Business, Jilin University, Changchun, China

**Keywords:** air quality, spatial distance, environmental affection, pro-environmental behavior, Chinese residents

## Abstract

Although environmental issues have attracted public attention, there are still many people unwilling to make behavioral changes to solve the problem, which makes promoting pro-environmental behavior become an interesting research topic. This study discusses the influence of air quality on the pro-environmental behavior of Chinese residents from the perspective of spatial distance, providing a theoretical basis and practical application for improving pro-environmental behavior. Through three experiments, this study reveals that air pollution within the local spatial distance could make residents more willing to conduct pro-environmental behavior. In addition, we also find that air pollution within local spatial distance would stimulate residents’ environmental affection and promote them to conduct pro-environmental behavior. That is, environmental affection plays a mediating role in the interactive effect of air quality and spatial distance on pro-environmental behavior.

## Introduction

The overexploitation of natural resources and the massive discharge of pollutants have brought great damage to the ecological environment, resulting in a series of severe environmental issues such as shortage of fresh water, land desertification, air pollution, water pollution, global warming, and acceleration of extinction of species (Steg and Vlek, 2009). The frequent occurrence of natural disasters like haze, acid rain, and sea level rise has not only restricted the sustainable development of economy but also seriously threatened health and life of human beings (Guo et al., 2020). Environmental issues have attracted great attention worldwide, and it is vital to find solutions to environmental degradation. Environmental issues caused by environmentally destructive human activities must be solved by human actions of environment protection (Bradley et al., 2020). Actively promoting pro-environmental behavior like green consumption, low-carbon travel, waste classification, and resources conservation may effectively reduce damage to ecological environment, enhance sustainable use of resources, and reduce degree of environmental pollution (Shi et al., 2017). Therefore, researchers have constructed and applied numerous theoretical frameworks to boost pro-environmental behavior, providing a solid theoretical basis. Some studies demonstrate that the theory of reasoned action (TRA) (Fishbein and Icek, 1975), the theory of planned behavior (TPB) (Ajzen, 1991), and the theory of responsible environmental behavior (Hines et al., 1987) can effectively explain the pro-environmental behavior, and that the individual’s personal factors such as attitude, subjective norms, and perceived behavior control determine one’s pro-environment behaviors. Many researchers also hold that individual norm, the sense of consequence, attribution of responsibility, and values are important determinants of pro-environmental behavior, explaining it through the norm-activation model (NAM) (Schwartz, 1977) and the value-belief-norm theory (VBN theory) (Stern et al., 1999), and begin to notice the influence of economic and social conditions and other external factors. With the increasing awareness of environmental issues, current studies pay a growing attention to the determinants of pro-environmental behavior (Geng et al., 2016; Wang et al., 2019).

Although scholars have had in-depth discussions on factors affecting pro-environmental behavior and its promoting mechanism, research concerning effects of contextual factors on pro-environmental behavior is relatively insufficient, and impacts of ecological environment conditions, say, air quality, are seldom considered. With the development of China’s economy, industrial growth, and advancing urbanization, air quality continues to deteriorate when acid rain, haze, and other harsh weather conditions occur more frequently, seriously affecting and endangering people’s health and daily life (Qiu, 2014). Air quality has also been widely explored by scholars. However, most of the research mainly focuses on its causes (Ramanathan et al., 2001) and the effects on residents’ health (Rajper et al., 2018), with only a few focusing on the influence of air quality on residents’ behavior. For instance, Sun J. et al. (2019) employed the two-way fixed effect panel model and concluded that as the public awareness of haze improved, high-level haze concentration may reduce residents’ domestic travel. Zhang et al. (2019) illustrated that the haze pollution perception would increase residents’ willingness of green consumption. Air pollution caused by human activities is a serious threat to human life and health, but how air quality affects residents’ pro-environmental behavior remains to be further discussed.

Air quality is the signal given by the ecological environment to residents, making residents have a perception of the current environmental conditions (Shi and He, 2012), and then affecting their behavior (Borbet et al., 2018). Air pollution would make people perceive the deterioration of the environment and then generate environmental affection such as worry, anxiety, or self-reproach, so as to be willing to conduct pro-environmental behavior. However, many people, even aware of existing environmental issues, are still unwilling to make behavioral changes to protect the environment (Xu et al., 2018). This study suggests that spatial distance from air pollution may complicate the process. The spatial distance from air pollution would greatly affect residents’ cognition of and affection for environmental conditions and then affect their subsequent behavior (Spence et al., 2012). Accordingly, this study designed three experiments to explore the influence of air quality on pro-environmental behavior at different spatial distances and to demonstrate the mediating role of environmental affection. The findings of this study would advance current understandings of the relationship between air quality and residents’ pro-environmental behavior from the perspective of spatial distance and provide practical applications for promoting residents’ pro-environmental behavior.

## Pro-Environmental Behavior

Pro-environmental behavior is also known as environment-friendly behavior, environmental responsible behavior, sustainable behavior, etc. (Kiatkawsin and Han, 2017). It refers to behavior that would reduce damage to the environment or be beneficial to the environment as much as possible (Steg and Vlek, 2009). From the perspective of environmental behavior science, scholars divide pro-environmental behavior into two types, namely, public pro-environmental behavior (such as becoming an active environmental citizen, supporting environmental policies, and joining in environment protection organizations) and private pro-environmental behavior (such as purchasing, using, and handling environmentally beneficial products or services by individuals or families) (Dietz et al., 1998; Stern, 2000). Compared with public pro-environmental behavior, private pro-environmental behavior requires less time and energy and is easier to do and to keep. Moreover, public pro-environmental behavior needs higher environmental awareness. The residents first consciously regulate their private sector behavior and become eco-friendly in their daily behavior. After that, they would further engage in all kinds of social environment protection activities and make more contributions to environmental protection.

In order to promote individuals to actively practice pro-environmental behavior, scholars have constructed many influential frameworks to explore antecedents of pro-environmental behavior. TRA (Fishbein and Icek, 1975) and TPB (Ajzen, 1991) discuss the predictive power of individual psychological variables, such as attitude, subjective norms, perceived behavior control, and behavioral intention, on behavior, while the influence of external situational variables is less considered. On this basis, Hines et al. (1987) added variables of responsibility, control point, skills, knowledge, economic conditions, and social pressure to propose a responsible environmental behavior model. Although they put forward that contextual variables would directly affect behavior, what they mainly focused on was still the impact of individual’s internal factors on the behavior. In addition, the NAM (Schwartz, 1977) and VBN theory (Stern et al., 1999) believe that individual norm, consequence consciousness, responsibility attribution, and values could predict individual altruistic behavior. Although these theories provide new mentality to the research of pro-environmental behavior, they still focus on the influence of individual’s internal motivations, while impacts of contextual factors have not been discussed systematically.

Based on the above theories, scholars have carried out in-depth exploration and discussions on determinants of pro-environmental behavior from two aspects of individual psychological characteristics and contextual factors. Studies of individual psychological characteristics affecting pro-environmental behavior are mainly on three ways of personality traits, cognition, and affection. First, scholars discuss the impacts of the personality traits, such as educational levels (Borbet et al., 2018), nature connectivity (Dutcher et al., 2007), values (Marshall et al., 2019), and mindfulness (Barbaro and Pickett, 2016), on pro-environmental behavior and reveal that people with certain pro-environmental or pro-social psychological traits are more willing to engage in pro-environmental behavior (Groot and Steg, 2008). Secondly, cognitive factors, such as perceived benefits and costs (Fritsche et al., 2010), could have greatly affected pro-environmental behavior. It is believed that individuals may make rational decisions based on the cognitive factors. In addition, some studies focus on the role of affection in predicting pro-environmental behavior, such as feelings of pride (Bissing-Olson et al., 2016), attitudes (Sun et al., 2020), environmental concern (Landry et al., 2018), and place attachment (Vaske and Kobrin, 2001), and put forward that affective factors have a strong explanatory power for pro-environmental behavior (Kim et al., 2018).

In fact, the motivating effects of individual psychological characteristics on pro-environmental behavior would be greatly influenced by external conditions (Guo et al., 2019). Therefore, scholars have further explored external factors influencing pro-environmental behavior from aspects of society, culture, media, and policy. Previous studies found that external situational variables, including social norms (Nolan et al., 2008) and expectations (Collado et al., 2017), public media (Sun Y. et al., 2019; Zhou et al., 2019), and governmental enforcement (Haddad, 2015), could play an important role in promoting pro-environmental behavior. It has thus expanded the research field of pro-environmental behavior and proposed new research ideas and directions for further exploring external factors affecting pro-environmental behavior in the future.

Although the existing research has discussed various inter-personal and contextual factors predicting pro-environmental behavior, there are still some limitations. First of all, most research still focuses on the influence of psychological variables on pro-environmental behavior, while the discussion of contextual variables is insufficient, and impacts of ecological environment are rarely considered. Secondly, current research on pro-environmental behavior is mostly from a single perspective of psychological or contextual factors, few of which consider comprehensively both external and internal factors. Furthermore, the underlying mechanisms of contextual variables affecting pro-environmental behavior remain to be further discussed. Therefore, this study explores the influence of air quality as an ecological environment condition on pro-environmental behavior in combination with spatial distance and discusses further the internal psychological mechanism from the perspective of environmental affection.

## Air Quality and Spatial Distance

Air pollution refers to the phenomenon that a large number of pollutants gather in the air and reach a certain level of concentration due to human activities or natural disasters. People usually use the quality of air to evaluate the degree of air pollution. Poor air quality may cause extreme weather such as haze, acid rain, and greenhouse effects, posing a serious threat to human life and health. The International Agency for Research on Cancer (IARC) of the World Health Organization (WHO) has rated outdoor air pollution as carcinogen. Some studies have shown that air quality would affect residents’ preference for environmentally friendly behavior, and air pollution would urge residents to engage in a variety of environmentally beneficial behaviors (Oltra et al., 2017). When air quality is low, the haze weather occurs as a signal of air pollution and could give residents more direct feeling about current environment condition. People would perceive a higher level of environmental risk and thus make environmentally friendly behavior decisions out of risk-averse motive (Zhang et al., 2019). However, some scholars do not believe that air pollution makes everyone pay more attention to environmental issues (Hatfield and Job, 2001). Many people remain indifferent to air pollution and would not have strong emotional response or behavioral intention (Bord et al., 2000; Gurajala et al., 2019). Residents’ different responses to the change of air quality may result from different spatial distance, so we cannot consider only impacts of air quality on pro-environmental behavior, but also the spatial distance between residents and the place where air pollution occurs.

Spatial distance, one of the dimensions of psychological distance, refers to the perception of distance or proximity of the target place with reference to the current location of the individual (Trope and Liberman, 2010). Previous research has shown that there are correlations between the four dimensions of psychological distance (temporal, spatial, social distance, and uncertainty). That is, the influence on one dimension will also affect the others (Bar-Anan et al., 2007). Psychological distance is widely used in the field of behavioral decision making and is believed to greatly influence people’s cognition, affection, and response to the environment (Liberman and Trope, 2008). In the field of ecological environment, although people are aware of global environmental issues, they are still unwilling to make behavioral changes from themselves because they think them far away (Xu et al., 2018). Meanwhile, some studies believe that people would be more willing to engage in pro-environmental behavior when the environmental issues become proximal to them. For example, Spence et al. (2012) explored impacts of psychological distance on sustainable behavior regarding environmental issues in four dimensions of time, space, society, and probability, and concluded that the shorter the psychological distance was, the higher people’s anxiety over environmental problems was, and the more likely they were to conduct sustainable behavior.

Air pollution in faraway places makes people feel powerless about their action and means that the impact of pro-environmental behavior remains uncertain and distant. They think that their pro-environmental behavior will not improve the phenomenon, and their destructive behavior will not worsen it (Dilling, 2007; Schill and Shaw, 2016). If people believe that the change of air quality is far away in space, they will not easily get worried about environment or health problems and will not take responsibility for environment on initiative, so it is not easy for them to take environmentally friendly actions (Sacchi et al., 2016; Wu and Geng, 2019). On the contrary, when change of air quality occurs in residents’ surroundings, their perception of environmental risks and worry about environmental problems grow greatly and their empathy for environmental problems will promote pro-environmental activities (Dong et al., 2019). On the one hand, low air quality makes residents aware of the serious air pollution issue (Wu and Geng, 2019). Therefore, when facing air pollution at close range, people may try to reduce air pollution by some behaviors that have direct effects on it like using public transportation instead of a private car. In addition, they would also adopt some behaviors that have indirect effects on air pollution, such as purchasing green products and participating in afforestation. On the other hand, air pollution also makes residents have a clearer understanding of the overall ecological environment and realize the seriousness and urgency of environmental issues. On this basis, people will make more efforts to improve the environmental status and have a higher willingness to conduct garbage classification, join in environmental protection organizations, advocate the concept of environmental protection, and other pro-environmental behavior. In addition, many people believe that personal actions can have an impact on the environment and that human activities are one of the causes of environmental issues (Pidgeon et al., 2008; Spence et al., 2012). Environmental pollution in the vicinity reflects consequences of residents’ past behavior (Zhang et al., 2019). It will not only make people feel regretful and guilty over past destructive behavior but also arouse compensatory pro-environmental behavior in the future. It also communicates that residents’ pro-environmental behavior will help improve the environment in the future, so that people can gain confidence to change the *status quo* of environment pollution through pro-environmental behavior. Therefore, this study believes that within local spatial distance, low (vs. high) air quality would stimulate residents’ pro-environmental behavior. Thus, the following hypotheses are proposed:

**H1a**: Within local spatial distance, relative to high air quality, low air quality increases Chinese residents’ pro-environmental behavior.

**H1b**: There is no significant difference between low and high air quality on Chinese residents’ pro-environmental behavior within distant spatial distance.

## The Mediating Effect of Environmental Affection

Affection is a response to causal-specific stimuli, and it is the relatively stable physiological evaluation and experience (Agarwal and Malhotra, 2005). Existing research conceptually makes a simple distinction between affection, emotion, and feeling. Feeling is a transient response to a particular situation based on the mind or senses, whereas emotion is a stable response to a particular situation (Agarwal and Malhotra, 2005). Affection is similarly defined as a stable and contextual response. Scholars often think that affection and emotion are basically the same (Gustafson, 1984), but there is still a slight difference in their meanings. Compared with emotion, affection is more based on certain value orientation or inclination (Choi, 2018). To date, the academics have not reached a unified understanding of environmental affection. Basing on the definition of affection, environmental affection is defined here as a stable emotional experience of ecological conditions and environment-related behaviors based on the yearning and love for the ecological environment. It means that people would have a stable emotional response to situations based on ecological commitments, cognition, judgments of value, and so on.

The state of ecological environment is the objective basis and important external stimuli for arousing residents’ environmental affection, and air quality is one of the important indicators reflecting the state of ecological environment. We suggest that low air quality within short distance will promote environmental affection. On the one hand, people usually have a place attachment to their residence. If air quality in the local area is low, people will easily get worried about environment pollution. It will also make people perceive threats of environmental problems to their daily life and health and thus become concerned and anxious over environmental problems (Hokka et al., 1999; Panu, 2016). On the other hand, the polluted air makes people think of their own and other residents’ environmental behavior and generate a sense of shame and guilt for their own destructive behavior (Rees et al., 2015), as well as a sense of disgust for others’ destructive behavior. However, low air quality at long spatial distance cannot promote people to produce strong environmental affection. If air pollution occurs in a faraway area, people cannot have a clear cognition of air quality or environment pollution and cannot have environmental affection of concern or anxiety over environmental problems. Meanwhile, they would not associate air pollution with their own deeds, so they are less likely to form environmental affection of shame or guilt.

With the progress of research, scholars have gradually found the importance of affection in promoting residents’ pro-environmental behavior. Some scholars even believe that behavior is based more on affective response than on cognitive factors that only play a secondary role (Wang et al., 2013). Affection can direct behavior, and many scholars have proved the significant positive effects of environmental affection on pro-environmental behavior. Chan and Lau (2000) found that ecological affect could indirectly affect green purchase behavior. Mallett (2012) found that ecological guilt would motivate eco-friendly behavior intention and then increase the possibility of participation in environment protection behavior. Generation of environmental affection reflects residents’ psychological activities: Their anxiety over bad environment caused by air pollution and love for a good ecological environment will urge them to make environmentally friendly behavior decisions, while emotions of guilt, pride, disgust, or appreciation generated from past behavior will guide people to correct destructive behavior and prefer pro-environmental behavior.

This study has proposed that the interaction of air quality and spatial distance would affect pro-environmental behavior of residents, in which environmental affection would play a mediating role. According to the “extended knowledge-attitude-practice (EKAP) model” in the field of psychology, the process of behavioral change can be divided into the following stages: the individuals form cognition and judgment of external situations. Then, they produce affection based on the cognition, and such affection affects the individual’s will to mobilize behavioral change (Wang et al., 2013). When the area of air pollution is close to the individual, lower air quality will stimulate the residents to recognize and judge on environmental issues and past environmental behaviors. Residents produce environmental affection on the basis of cognition, such as anxiety over environmental problems, longing for a better environment, guilt of and disgust at destructive environmental behavior, and pride in and appreciation of pro-environmental behavior. Under the direction of a series of environmental affection, people will generate behavioral motivation to protect the environment and tend to engage in pro-environmental behavior. To sum up, this study proposes the following hypotheses:

**H2a**: Within local spatial distance, environmental affection plays a mediating role between air quality and pro-environmental behavior.

**H2b:** Within distant spatial distance, the mediating role of environmental affection does not exist.

**FIGURE 1:**
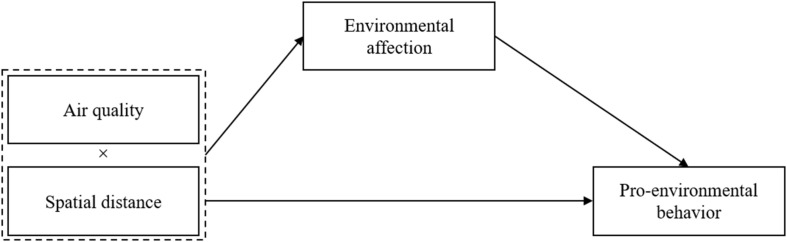
Framework.

To sum up, this study proposes a conceptual framework of impacts of air quality on residents’ pro-environmental behavior (Figure 1).

## Materials and Methods and Results

### Pretest

Pretest was divided into three parts: the selection of weather phenomena in the materials, the selection of cities in the materials, and the check of manipulation.

In the first part, we initially selected the weather phenomena representing low air quality. With reference to air quality evaluation indicators (SO_2_, NO_2_, PM_10_, PM_2_._5_, CO, O_3_, etc.), we selected seven air pollution phenomena released by the Ministry of Ecology and Environment of China, including acid rain, haze, greenhouse effect, and sulfuric acid smog, as alternative weather phenomena. The experiments require the subjects to be familiar with the selected air pollution phenomenon and have a clear understanding of the air quality it represents. Therefore, familiarity and perceptual air quality were taken as the criteria for screening. Fifty undergraduate students in Jilin University participated in this pretest and were asked to indicate (1) how familiar they are with each of the seven weather phenomena and (2) which level they ranked the air quality from each of the seven weather phenomena, by using a seven-point scale (1 = “very unfamiliar” and 7 = “very familiar;” 1 = “very low” and 7 = “very high”). Results showed that the participants were most familiar with haze weather (*M* = 6.68, *SD* = 0.47), followed by acid rain (*M* = 6.10, *SD* = 0.74), and the perceptual air quality of haze weather was the lowest (*M* = 1.58, *SD* = 0.64), followed by acid rain (*M* = 2.12, *SD* = 0.75). Based on these findings, haze weather and clear weather would be used to manipulate air quality in the following experiments.

The aim of the second part was to select two cities that could manipulate the spatial distance. Spatial distance refers to perception of the distance of the target location. Therefore, we selected cities according to the following criteria: (1) the cities have experienced haze weather; (2) the subjects could clearly perceive the spatial distance at a local/distant level. First, since the experiments would be conducted in Changchun, China, we decided to use Changchun as the local spatial distance. Next, we selected 10 famous cities with obvious geographical distance from Changchun as candidates, including Beijing, Shanghai, Guangzhou, London, Seoul, Paris, etc. Fifty undergraduate students in Jilin University participated in this pretest and rated the perceptual spatial distance to the 10 cities on a seven-point scale (1 = “very close” and 7 = “very far”). Results showed that the participants perceived a farthest spatial distance from London (*M* = 6.06, *SD* = 0.84), so we decided to use London and Changchun to manipulate spatial distance.

In the third part, we checked the manipulation material of air quality and spatial distance. In psychological experiments, text, images, or videos are often used as stimulus materials to induce specific responses, among which images and videos can play a more effective role through visual effects. Furthermore, compared with video, the image stimulus is easier to operate and response of the subjects is easier to observe. Therefore, we combined the haze and clear weather with the landmark buildings in Changchun and London and invited professional designers to design four groups of still images A, B, C, and D as stimulus materials. Eighty undergraduates in Jilin University participated in this pretest and were randomly assigned into a 2 (Air quality: high vs. low) × 2 (Spatial distance: local vs. distant) between-subjects design. In each condition, participants were asked to browse the images above, respectively, and then rated the air quality and spatial distance that they perceived from the images on a seven-point scale. A *t*-test for the air quality manipulation check revealed that the perceived air quality in the low air quality condition was significantly lower than that in the high air quality condition, M_low_ = 1.60, *SD* = 0.59 vs. M_high_ = 6.40, *SD* = 0.63, *t*(78) = 35.08, *p* < 0.001, Cohen’s *d* = 7.94, which means the manipulation of air quality performed as intended. A *t*-test for the spatial distance manipulation check revealed that the perceptual spatial distance in the local spatial distance condition was significantly lower than that in the distant spatial distance condition, M_local_ = 1.85, *SD* = 0.80 vs. M_distant_ = 6.23, *SD* = 0.62, *t*(78) = 27.30, *p* < 0.001, Cohen’s *d* = 6.18, which means the manipulation of spatial distance performed as intended.

### Experiment 1

To test H1a and H1b, Experiment 1 was designed to provide an examination of how air quality affects pro-environmental behavior intention within different spatial distance.

#### Participants and Procedure

Two hundred ten undergraduates in Changchun participated in the experiment (M_age_ = 20.42, *SD* = 1.43; 51.9% female, *N* = 107). Before the actual experiment procedure, each participant was informed that they would be shown several images and then completed a questionnaire. Each participant was given a cash reward of 5 yuan after completing the experiment. To eliminate the interference of basic mood, participants were asked to report their basic mood (e.g., happy, sad, angry, fear, etc.) on a seven-point scale. We removed four participants who had an obvious mood tendency.

Two hundred six participants who completed the mood test were randomly assigned to a 2 (Air quality: high vs. low) × 2 (Spatial distance: local vs. distant) between-subjects experimental design. We manipulated the air quality and spatial distance by presenting images that featured landmark buildings in either Changchun or London on an either clear or haze day, respectively. Participants were asked to look at the images for 5 s and then report the air quality and spatial distance that they perceived from the images as a manipulation check.

Next, participants completed a pro-environmental behavior intention scale, based on the Chinese General Social Survey (CGSS), which is the earliest project to do a national, comprehensive, and continuous academic survey. The scale contains a total of 10 items, including 5 items in the private dimension, such as “garbage classification,” and 5 items in the public dimension, such as “donation for environmental protection” (Table 1). Participants rated their intention to engage in these behaviors on a seven-point scale (1 = “very unwilling” and 7 = “very willing”). We summed scores on the 10 items to measure pro-environmental behavior intention and summed the scores on the 5 items from the private dimension to measure private pro-environmental behavior intention and the 5 items from the public dimension to measure public pro-environmental behavior intention. At the end of the experiment, participants indicated their age and gender.

**TABLE 1:** Scales of pro-environmental behavior intention and environmental affection.

**Pro-environmental behavior intention**
Garbage classification
Discuss environmental issues with friends and relatives
Bring your own basket (bag) for grocery shopping
Purchase green products
Take public transport instead of private cars
Contribute to environmental protection
Participate in environmental publicity organized by the government and the community
Participate in environmental protection activities organized by non-governmental organizations
Attend the maintenance of trees or green space at your own expense
Participate in complaints and appeal activities to solve environmental issues
**Environmental affection**
I am concerned about the environmental pollution issue.
I am worried about the environmental pollution issue.
I am angry about the environmental pollution issue.
I love the environment without pollution.
I aspire to the environment without pollution.
I cherish the environment without pollution.
I am disgusted by the destruction of the environment.
I despise the destruction of the environment.
The destruction of the environment infuriates me.
I am ashamed of the destruction of the environment.
I feel guilty about destroying the environment.
I feel sad about the destruction of the environment.
I praise the act of protecting the environment.
I appreciate the act of protecting the environment.
I respect the protection of the environment.
I am gratified by the act of protecting the environment.
I am delighted with the act of protecting the environment.
I am proud of the act of protecting the environment.

#### Results

##### Reliability and validity analysis

Cronbach’s alpha reliability for “pro-environmental behavior intention” equaled 0.87, greater than 0.7, indicating that the reliability of the measurement was acceptable. Average variance extracted (AVE) of “pro-environmental behavior intention” was 0.54, surpassing the acceptable level of 0.50, indicating a satisfactory level of convergent validity.

##### Manipulation checks

To begin, we examined the perceptual air quality of the images used in the experiment. A *t*-test was used to compare perception of the air quality of the images in high air quality conditions and that in low air quality conditions. Results revealed that participants in the high air quality conditions perceived higher air quality (M_high_ = 6.08, *SD* = 0.88) than participants in the low air quality conditions [M_low_ = 1.52, *SD* = 0.50, *t*(204) = 45.62, *p* < 0.001, Cohen’s *d* = 6.39]. We also examined the perceptual spatial distance of the images used in the experiment. A *t*-test was used to compare perception of the spatial distance of the images in the local spatial distance conditions and that in the distant spatial distance conditions. Results revealed that participants in the local spatial distance conditions perceived closer spatial distance (M_local_ = 6.45, *SD* = 0.61) than participants in distant spatial distance conditions [M_distant_ = 1.75, *SD* = 0.57, *t*(204) = 57.20, *p* < 0.001, Cohen’s *d* = 8.02]. Thus, the manipulation of air quality and spatial distance performed as intended.

##### Main effect analysis

**FIGURE 2:**
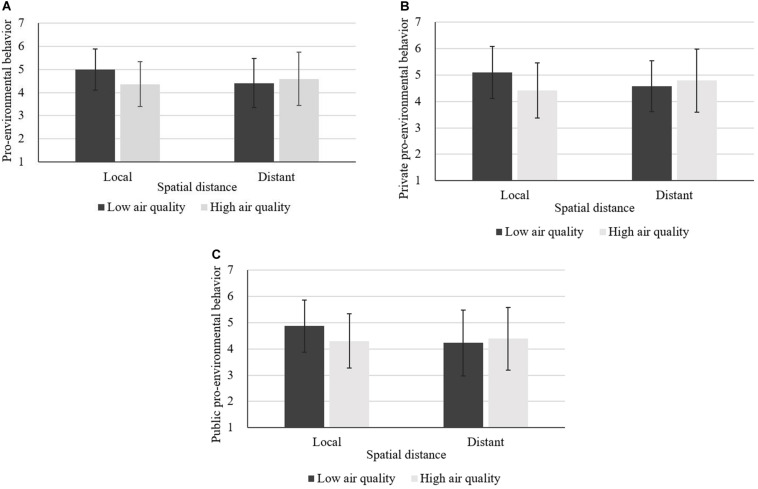
**(A–C)** Main effect analysis of Experiment 1.

An analysis of variance (ANOVA) was conducted on pro-environmental behavior intention. Results revealed an interaction effect between air quality and spatial distance [*F*(1,202) = 8.03, *p* = 0.005, η^2^ = 0.038], while the main effects of air quality [*F*(1,202) = 2.33, *p* = 0.128] and spatial distance [*F*(1,202) = 1.51, *p* = 0.220] on pro-environmental behavior intention were not significant. The results of the simple effect analysis showed that, when the spatial distance was at local level, compared with high air quality (M_high_ = 4.36, *SD* = 0.97), low air quality led to higher pro-environmental behavior intention (M_low_ = 4.99, *SD* = 0.89), *F*(1,203) = 9.47, *p* = 0.002, η^2^ = 0.044. However, when the spatial distance was long, air quality had no significant effect on pro-environmental behavior intention (M_low_ = 4.41, *SD* = 1.06, M_high_ = 4.59, *SD* = 1.15), *F* < 1 (Figure 2A).

Next, we used a paired-samples *t*-test to compare private and public pro-environmental behavior intentions. Results showed that private pro-environmental behavior intention and public pro-environmental behavior intention were positively correlated (*r* = 0.78, *p* < 0.001) and that participants rated higher intention to engage in private (vs. public) pro-environmental behavior (M_private_ = 4.73, *SD* = 1.07, M_public_ = 4.45, *SD* = 1.15), *t*(205) = 5.45, *p* < 0.001.

We also conducted a multivariate analysis of variance (MANOVA) on private and public pro-environmental behavior intention to further examine the interaction effect between air quality and spatial distance on private and public pro-environmental behavior intention. Results showed that the interaction between air quality and spatial distance had an effect on both private and public pro-environmental behavior intention, and the interaction effect was greater on private pro-environmental behavior intention, *F*_private_(1,202) = 9.07, *p* = 0.003, η^2^ = 0.043, *F*_public_(1,202) = 5.52, *p* = 0.020, η^2^ = 0.027, whereas the main effects of air quality [*F*_air quality_(1,202) = 2.69, *p* = 0.102] and spatial distance (*F*_spatial distance_ < 1) on private pro-environmental behavior intention were not significant. The main effects of air quality [*F*_air quality_(1,202) = 1.56, *p* = 0.213] and spatial distance [*F*_spatial distance_(1,202) = 3.25, *p* = 0.073] on public pro-environmental behavior intention were not significant. The results of the simple effect analysis revealed that, when the spatial distance was at local level, air quality had a significant effect on both private [M_low_ = 5.10, *SD* = 0.98, M_high_ = 4.42, *SD* = 1.04, *F*(1,203) = 10.72, *p* = 0.001, η^2^ = 0.050] and public pro-environmental behavior intention [M_low_ = 4.87, *SD* = 1.00, M_high_ = 4.31, *SD* = 1.03, *F*(1,203) = 6.48, *p* = 0.012, η^2^ = 0.031]. However, as for the distant level of spatial distance, air quality had no significant effect on private (M_low_ = 4.59, *SD* = 0.96, M_high_ = 4.79, *SD* = 1.19, *F* < 1) or public pro-environmental behavior intention (M_low_ = 4.22, *SD* = 1.26, M_high_ = 4.39, *SD* = 1.20, *F* < 1) (Figures 2B,C).

#### Discussion

In support of H1a and H1b, Experiment 1 demonstrated that low air quality promotes pro-environmental behavior intention when the spatial distance is at local level. Notably, air quality has no significant effect on pro-environmental behavior intention when the spatial distance is long. Furthermore, the findings of Experiment 1 also suggested that, compared with the public domain, participants are more likely to engage in private pro-environmental behavior, and the interactive effect of air quality and spatial distance on private pro-environmental behavior intention is greater. Although the results of Experiment 1 were in line with expectations, the student samples still had some limitations. As such, we conducted Experiment 2 to replicate the results of Experiment 1 with non-student samples and to further verify the mediating effect of environmental affection.

### Experiment 2

To test H2a and H2b, we designed Experiment 2 to examine the mediating role of environmental affection.

#### Participants and Procedure

The participants in Experiment 1 were undergraduate students whose ages were between 18 and 23, so the results were questionable for other age groups. Furthermore, compared with local residents, undergraduate students may only live in Changchun for a relatively short time. To generalize our findings, Experiment 2 changed the participants from student samples to non-student samples. Two hundred thirty-six adult residents in Changchun, aged between 18 and 40, participated in the experiment (M_age_ = 29.09, *SD* = 6.46, 50.6% female, *N* = 117). Before the actual experiment procedure, each participant was informed that they would be shown several images and then complete a questionnaire. Each participant was given a cash reward of 5 yuan after completing the experiment. To eliminate the interference of basic mood, participants were asked to report their basic mood. We removed five samples with an obvious mood tendency.

Two hundred thirty-one participants with no obvious mood tendency were randomly assigned to a 2 (Air quality: high vs. low) × 2 (Spatial distance: local vs. distant) between-subjects experimental design. We used some images to manipulate air quality and spatial distance as we did in Experiment 1, but there were also some differences as we changed the landmarks in the images. Participants in the four experiment groups were shown some images that were very similar to that in Experiment 1, respectively. Participants were asked to look at the images for 5 s. Next, as manipulation check, participants also reported their perceptual air quality and perceptual spatial distance the same as in Experiment 1.

After that, participants indicated their environmental affection and pro-environmental behavior intention. The items we adopted to measure environmental affection were based on previous literature on people’s various affection for the environment (Maloney et al., 1975; Koenig-Lewis et al., 2014) including “worried,” “disgust,” “guilt,” “love,” “praise,” and “pride,” and the scale was slightly modified on the basis of the context of pro-environmental behavior. Participants reported their environmental affection by rating 18 items, such as “I am worried about environmental pollution issue,” “I am disgusted by environmental destruction behavior,” “I feel guilty about harming the environment,” etc. (1 = “strongly disagree,” 7 = “strongly agree;” Table 1). We used the same 10 items as that used in Experiment 1 to measure pro-environmental behavior intention. Participants also indicated their age and gender.

#### Results

##### Reliability and validity analysis

Cronbach’s alpha reliability for “environmental affection” equaled 0.94 and that for “pro-environmental behavior intention” equaled 0.89, both greater than the acceptable level (0.7). AVEs of “environmental affection” and “pro-environmental behavior intention” equaled 0.54 and 0.46, which approximated to 0.50 or surpassed 0.50, and the CRs of the two constructs were 0.93 and 0.89, indicating an acceptable level of convergent validity. The correlation between environmental affection and pro-environmental behavior intention was 0.64 (*p* < 0.001), which was below the smallest square root of the AVE (0.68), indicating that the discriminant validity of the measurements was acceptable.

##### Manipulation checks

Firstly, we conducted a *t*-test for air quality manipulation check. Results showed that the perceptual air quality of participants in high air quality conditions (M_high_ = 6.00, *SD* = 0.66) was significantly higher than that in low air quality conditions (M_low_ = 2.07, *SD* = 0.63), *t*(229) = 46.23, *p* < 0.001, Cohen’s *d* = 6.11. We also conducted a *t*-test for spatial distance manipulation check. Results showed that the perceptual spatial distance of participants in distant spatial distance conditions (M_distant_ = 6.32, *SD* = 0.54) was significantly higher than that in local spatial distance conditions (M_local_ = 1.61, *SD* = 0.71), *t*(229) = 56.69, *p* < 0.001, Cohen’s *d* = 7.52. The air quality and spatial distance manipulation performed as intended.

##### Main effect analysis

**FIGURE 3:**
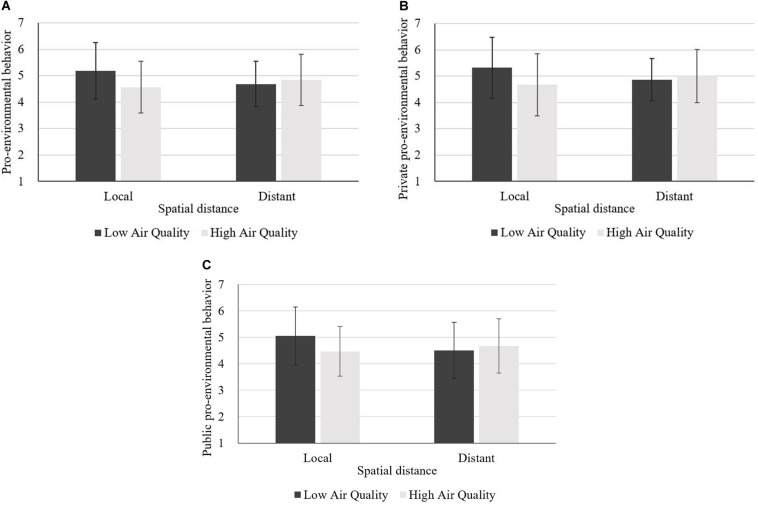
**(A–C)** Main effect analysis of Experiment 2.

We conducted an ANOVA on pro-environmental behavior intention to confirm the findings of Experiment 1. The results revealed an interactive effect of air quality and spatial distance on pro-environmental behavior intention, *F*(1,227) = 9.11, *p* = 0.003, η^2^ = 0.039, whereas the main effects of air quality [*F*(1,227) = 3.30, *p* = 0.070] and spatial distance (*F* < 1) on pro-environmental behavior intention were not significant. Contrasts showed that, when the spatial distance was at local level, low (vs. high) air quality led to higher pro-environmental behavior intention (M_low_ = 5.19, *SD* = 1.07, M_high_ = 4.57, *SD* = 0.98), *F*(1,228) = 11.28, *p* = 0.001, η^2^ = 0.050. However, when the spatial distance was long, air quality had no significant effect on pro-environmental behavior intention (M_low_ = 4.69, *SD* = 0.86, M_high_ = 4.84, *SD* = 0.98), *F* < 1 (Figure 3A).

Next, to examine the findings of Experiment 1 that the intention of private pro-environmental behavior is higher than that of public pro-environmental behavior, we conducted a paired-samples *t*-test to compare private and public pro-environmental behavior intentions. Results revealed that private and public pro-environmental behavior intentions were positively correlated (*r* = 0.75, *p* < 0.001) and that participants had higher intention to engage in private pro-environmental behavior (M_private_ = 4.97, *SD* = 1.07, M_public_ = 4.68, *SD* = 1.05), *t*(230) = 5.85, *p* < 0.001.

We also conducted a MANOVA to further examine the interactive effect of air quality and spatial distance on private and public pro-environmental behavior intention. Results showed that the interaction between air quality and spatial distance had an effect on both private and public pro-environmental behavior intention, and this interactive effect on private pro-environmental behavior intention was greater, *F*_private_(1,227) = 8.13, *p* = 0.005, η^2^ = 0.035 vs. *F*_public_(1,227) = 7.67, *p* = 0.006, η^2^ = 0.033, while the main effects of air quality [*F*_air quality_(1,227) = 3.50, *p* = 0.063] and spatial distance (*F*_spatial distance_ < 1) on private pro-environmental behavior intention were not significant. The main effects of air quality [*F*_air quality_(1,227) = 2.29, *p* = 0.132] and spatial distance [*F*_spatial distance_ (1,227) = 1.58, *p* = 0.211] on public pro-environmental behavior intention were not significant. Results of the simple effect analysis revealed that, when the spatial distance was at local level, air quality had a significant effect on both private [M_low_ = 5.33, *SD* = 1.16, M_high_ = 4.67, *SD* = 1.88, *F*(1,228) = 11.40, *p* = 0.001, η^2^ = 0.048] and public pro-environmental behavior intention [M_low_ = 5.05, *SD* = 1.10, M_high_ = 4.47, *SD* = 0.94, *F*(1,228) = 9.40, *p* = 0.002, η^2^ = 0.039]. As for the distant level of spatial distance, air quality had no significant effect on private (M_low_ = 4.87, *SD* = 0.80, M_high_ = 5.00, *SD* = 1.01, *F* < 1) or public pro-environmental behavior intention (M_low_ = 4.51, *SD* = 1.06, M_high_ = 4.68, *SD* = 1.03, *F* < 1) (Figures 3B,C).

##### Mediating effect analysis

We conducted an ANOVA with air quality and spatial distance on environmental affection. Results revealed an interactive effect of air quality and spatial distance on environmental affection, *F*(1,227) = 4.20, *p* = 0.042, η^2^ = 0.018. The main effects of air quality [*F*_air quality_(1,227) = 3.85, *p* = 0.051] and spatial distance (*F*_spatial distance_ < 1) on environmental affection were not significant. Results of the simple effect analysis revealed that, when the spatial distance was at local level, low (vs. high) air quality led to higher environmental affection (M_low_ = 5.35, *SD* = 1.09, M_high_ = 4.80, *SD* = 1.22), *F*(1,228) = 8.24, *p* = 0.004, η^2^ = 0.035. However, when the spatial distance was long, air quality had no significant effect on environmental affection (M_low_ = 4.99, *SD* = 0.81, M_high_ = 5.00, *SD* = 1.05), *F* < 1.

**FIGURE 4:**
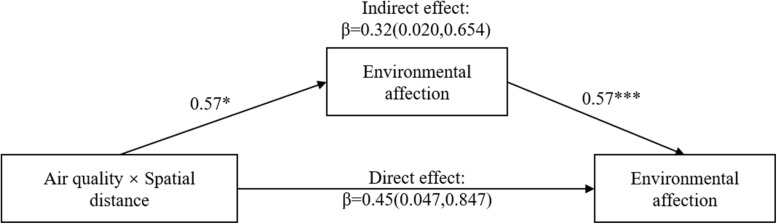
Mediation analysis of Experiment 2. *p < 0.05; ***p < 0.001.

We used the PROCESS macro to test the mediating role of environmental affection. We selected model 8 and conducted a bootstrap at the 95% confidence interval from 5000 bootstrap samples (Hayes, 2013). As shown in Figure 4, the direct effect of the interaction between air quality and spatial distance, after controlling for environmental affection, was found to be β = 0.45 (LLCI = 0.047, ULCI = 0.847). The indirect effect of the highest-order interaction was found to be β = 0.32, and the bias-corrected 95% confidence interval did not include zero (LLCI = 0.020, ULCI = 0.654), demonstrating that environmental affection mediated the interactive effect of air quality and spatial distance on pro-environmental behavior intention. Furthermore, as presented in Table 2, the results of the analysis for the conditional direct effects of air quality on pro-environmental behavior intention at values of spatial distance revealed that, when the spatial distance was at local level, air quality had a significant effect on pro-environmental behavior intention (β = 0.30, *t* = 2.09, *p* = 0.038), whereas within distant spatial distance, air quality had no significant effect on pro-environmental behavior intention (β = -0.15, *t* = -1.02, *p* = 0.307). In addition, the results of the analysis for the conditional indirect effects of air quality on pro-environmental behavior intention at values of spatial distance revealed that, when the spatial distance was at local level, the mediating effect of environmental affection was found to be β = 0.32, and the bias-corrected 95% confidence interval did not include zero (LLCI = 0.087, ULCI = 0.570), whereas when the spatial distance was at distant level, the mediating effect of environmental affection was found to be non-significant (LLCI = -0.207, ULCI = 0.192).

**TABLE 2:** Conditional direct and indirect effects in Experiment 2.

	Spatial distance	Effect	SE	*t*	*p*	LLCI	ULCI
Direct effect	Local	0.30	0.14	2.09	0.038	0.017	0.584
	Distant	–0.15	0.14	–1.02	0.307	–0.429	0.136
Indirect effect	Local	0.32	0.12			0.087	0.570
	distant	–0.01	–0.10			–0.207	0.192

#### Discussion

Experiment 2 re-examined the interactive effect between air quality and spatial distance on pro-environmental behavior intention with non-student samples, increasing the stability of the experimental results. Experiment 2 again proved that participants showed higher intention to private (vs. public) pro-environmental behavior, and the interactive effect of air quality and spatial distance on private (vs. public) pro-environmental behavior intention was greater. Furthermore, in support of H2a and H2b, Experiment 2 showed that environmental affection mediated the interactive effect of air quality and spatial distance on pro-environmental behavior intention. Notably, only when the spatial distance is at local level does low air quality trigger greater environmental affection, leading to a higher intention to engage in pro-environmental behavior. In both Experiment 1 and Experiment 2, London was selected to manipulate distant spatial distance. However, there are still some obvious differences between London and Changchun, such as nationality and culture, which may contribute to certain disturbances. Therefore, Experiment 3 was conducted to re-examine the results in Experiment 2 by selecting Guangzhou, a city in China that is also at a distinct geographical distance from Changchun, to manipulate spatial distance with the purpose of improving the robustness of the experiment.

### Experiment 3

#### Participants and Procedure

To generalize our findings, Experiment 3 used Guangzhou, a city in China, to manipulate the distant spatial distance, according to the requirements of the experiment: (a) haze phenomenon exists; (b) a well-known city familiar to people; (c) there is an obvious geographical distance from Changchun. Moreover, Guangzhou and Changchun are both provincial capitals located in China, which could reduce the interference of national and cultural differences to some extent. One hundred eighty adult residents in Changchun, aged between 18 and 40, participated in the experiment (M_age_ = 28.90, *SD* = 6.74, 51.1% female, *N* = 92). Before the actual experiment procedure, each participant was informed that they would be shown several images and then complete a questionnaire. Each participant was given a cash reward of 5 yuan after completing the experiment. To eliminate the interference of basic mood, participants were asked to report their basic mood. Then, we removed three samples with an obvious mood tendency.

One hundred seventy-seven participants with no obvious mood tendency were randomly assigned to a 2 (Air quality: high vs. low) × 2 (Spatial distance: local vs. distant) between-subjects experimental design. We manipulated the air quality and spatial distance by presenting images that featured landmark buildings in either Changchun or Guangzhou on an either clear or haze day, respectively. Participants were asked to look at the images for 5 s. Next, as manipulation check, participants also reported their perceptual air quality and perceptual spatial distance the same as in Experiment 2.

After that, participants indicated their environmental affection and pro-environmental behavior intention. We used the same measurements as that used in Experiment 2 to measure environmental affection and pro-environmental behavior intention. Participants also indicated their age and gender.

#### Results

##### Reliability and validity analysis

Cronbach’s alpha reliability for “environmental affection” equaled 0.94 and that for “pro-environmental behavior intention” equaled 0.91, both greater than the acceptable level (0.7). AVEs of “environmental affection” and “pro-environmental behavior intention” equaled 0.56 and 0.50, which equaled or surpassed 0.50, and the CRs of the two constructs were 0.93 and 0.91, indicating that there was convergent validity. The correlation between environmental affection and pro-environmental behavior intention was 0.31 (*p* < 0.001), which was below the smallest square root of the AVE (0.71), indicating that the discriminant validity of the measurements was acceptable.

##### Manipulation checks

Firstly, we conducted a *t*-test for air quality manipulation check. Results showed that the perceptual air quality of participants in high air quality conditions (M_high_ = 6.03, *SD* = 0.64) was significantly higher than that in low air quality conditions (M_low_ = 2.04, *SD* = 0.62), *t*(175) = 42.07, *p* < 0.001, Cohen’s *d* = 6.36. We also conducted a *t*-test for spatial distance manipulation check. Results showed that the perceptual spatial distance of participants in distant spatial distance conditions (M_distant_ = 6.33, *SD* = 0.56) was significantly higher than that in local spatial distance conditions (M_local_ = 1.69, *SD* = 0.73), *t*(167) = 47.31, *p* < 0.001, Cohen’s *d* = 7.31. The air quality and spatial distance manipulation performed as intended.

##### Main effect analysis

**FIGURE 5:**
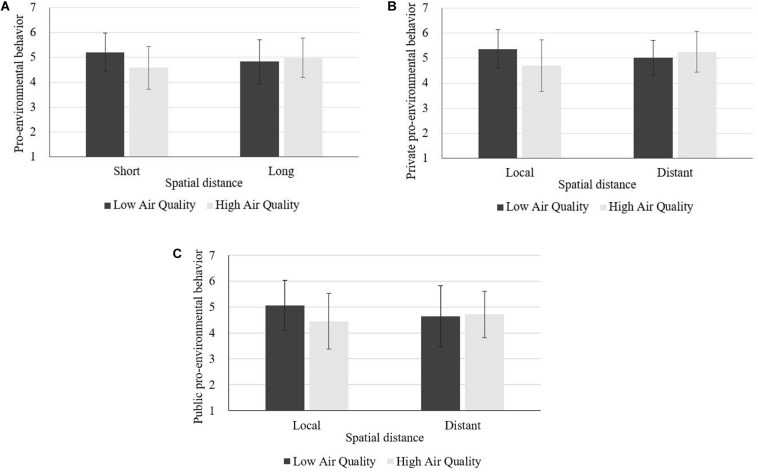
**(A–C)** Main effect analysis of Experiment 3.

The results of ANOVA on pro-environmental behavior intention revealed an interactive effect between air quality and spatial distance on pro-environmental behavior intention, *F*(1,173) = 9.80, *p* = 0.002, η^2^ = 0.054, while the main effects of air quality [*F*(1,174) = 3.77, *p* = 0.054] and spatial distance (*F* < 1) on pro-environmental behavior intention were not significant. Contrasts showed that, when the spatial distance was at local level, low (vs. high) air quality led to higher pro-environmental behavior intention (M_low_ = 5.21, *SD* = 0.77, M_high_ = 4.58, *SD* = 0.86), *F*(1,174) = 12.71, *p* < 0.001, η^2^ = 0.068. However, when the spatial distance was long, air quality had no significant effect on pro-environmental behavior intention (M_low_ = 4.84, *SD* = 0.88, M_high_ = 4.98, *SD* = 0.79), *F* < 1 (Figure 5A).

Next, we conducted a paired-samples *t*-test to compare private and public pro-environmental behavior intentions. Results revealed that private and public pro-environmental behavior intentions were positively correlated (*r* = 0.58, *p* < 0.001) and that participants had higher intention to engage in private (vs. public) pro-environmental behavior (M_private_ = 5.09, *SD* = 0.86, M_public_ = 4.72, *SD* = 1.05), *t*(176) = 5.50, *p* < 0.001.

We also conducted a MANOVA to further examine the interactive effect between air quality and spatial distance on private and public pro-environmental behavior intention. Results showed that the interaction between air quality and spatial distance had an effect on both private and public pro-environmental behavior intention, and this interactive effect on private pro-environmental behavior intention was greater, *F*_private_(1,173) = 12.78, *p* < 0.001, η^2^ = 0.069 vs. *F*_public_(1,173) = 4.47, *p* = 0.036, η^2^ = 0.025, while the main effects of air quality [*F*_air quality_(1,173) = 2.80, *p* = 0.096] and spatial distance (*F*_spatial distance_ < 1) on private pro-environmental behavior intention were not significant. The main effects of air quality [*F*_air quality_(1,173) = 3.07, *p* = 0.082] and spatial distance (*F* < 1) on public pro-environmental behavior intention were not significant. Results of the simple effect analysis revealed that, when the spatial distance was at local level, air quality had a significant effect on both private [M_low_ = 5.37, *SD* = 0.77, M_high_ = 4.71, *SD* = 1.03, *F*(1,174) = 13.45, *p* < 0.001, η^2^ = 0.072] and public pro-environmental behavior intention [M_low_ = 5.05, *SD* = 0.97, M_high_ = 4.45, *SD* = 1.07, *F*(1,174) = 7.45, *p* = 0.007, η^2^ = 0.041]. As for long spatial distance, air quality had no significant effect on private [M_low_ = 5.02, *SD* = 0.70, M_high_ = 5.26, *SD* = 0.82, *F*(1,174) = 1.78, *p* = 0.184] or public pro-environmental behavior intention (M_low_ = 4.66, *SD* = 1.17, M_high_ = 4.71, *SD* = 0.89, *F* < 1) (Figures 5B,C).

##### Mediating effect analysis

We conducted an ANOVA with air quality and spatial distance on environmental affection. Results revealed an interactive effect of air quality and spatial distance on environmental affection, *F*(1,173) = 4.42, *p* = 0.037, η^2^ = 0.025. The main effects of air quality [*F*_air quality_(1,173) = 1.64, *p* = 0.202] and spatial distance (*F*_spatial distance_ < 1) on environmental affection were not significant. Results of the simple effect analysis revealed that, when the spatial distance was at local level, low (vs. high) air quality led to higher environmental affection (M_low_ = 5.24, *SD* = 0.87, M_high_ = 4.72, *SD* = 1.09), *F*(1,174) = 5.73, *p* = 0.018, η^2^ = 0.032, whereas air quality had no significant effect on environmental affection when the spatial distance was at distant level (M_low_ = 4.81, *SD* = 0.78, M_high_ = 4.93, *SD* = 1.26), *F* < 1.

**FIGURE 6:**
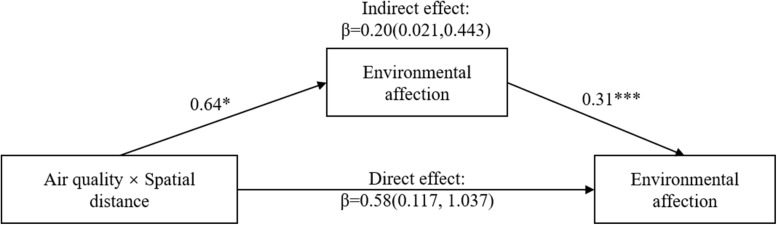
Mediation analysis of Experiment 3. *p < 0.05; ***p < 0.001.

To test the mediating role of environmental affection, we conducted a bootstrap at the 95% confidence interval from 5000 bootstrap samples by using the PROCESS macro (Model 8). As shown in Figure 6, the direct effect of the interaction between air quality and spatial distance, after controlling environmental affection, was found to be β = 0.58 (LLCI = 0.117, ULCI = 1.037). The indirect effect of the highest-order interaction was found to be β = 0.20, and the bias-corrected 95% confidence interval did not include zero (LLCI = 0.021, ULCI = 0.443), demonstrating that environmental affection mediated the interactive effect of air quality and spatial distance on pro-environmental behavior intention. Furthermore, as presented in Table 3, the results of the analysis for the conditional direct effects of air quality on pro-environmental behavior intention at values of spatial distance revealed that, when the spatial distance was at local level, air quality had a significant effect on pro-environmental behavior intention (β = 0.47, *t* = 2.81, *p* = 0.006), whereas air quality had no significant effect on pro-environmental behavior intention within distant spatial distance (β = -0.11, *t* = -0.67, *p* = 0.503). In addition, the results of the analysis for the conditional indirect effects of air quality on pro-environmental behavior intention at values of spatial distance revealed that, when the spatial distance was at local level, the mediating effect of environmental affection was found to be β = 0.16, and the bias-corrected 95% confidence interval did not include zero (LLCI = 0.033, ULCI = 0.334), whereas the mediating effect of environmental affection was found to be non-significant (LLCI = -0.188, ULCI = 0.098) when the spatial distance was at distant level.

**TABLE 3:** Conditional direct and indirect effects in Experiment 3.

	Spatial distance	Effect	SE	*t*	*p*	LLCI	ULCI
Direct effect	Local	0.47	0.17	2.81	0.006	0.140	0.798
	Distant	–0.11	0.16	–0.67	0.503	–0.427	0.210
Indirect effect	Local	0.16	0.08			0.033	0.334
	distant	–0.04	0.07			–0.188	0.098

#### Discussion

The results of Experiment 3 replicate the findings of Experiment 2. When facing the low air quality (vs. high air quality) at local spatial distance, residents reported a higher intention to engage in pro-environmental behavior, whereas at distant spatial distance, the difference between low and high air quality was not significant. Additionally, residents reported a higher intention to engage in private (vs. public) pro-environmental behavior. The interactive effect of air quality and spatial distance on private pro-environmental behavior intention was greater. Our moderated mediation analysis also revealed that the interactive effect of air quality and spatial distance influenced environmental affection and, in turn, intention to engage in pro-environmental behavior.

## Discussion and Conclusion

In this study, three experiments were designed to verify the interactive effect of air quality and spatial distance on Chinese residents’ pro-environmental behavior intention and to examine the mediating effect of environmental affection. Based on the results, the following conclusions were drawn. First, air quality and spatial distance have significant interactive effect on residents’ pro-environmental behavior intention. That is, within local spatial distance, low (vs. high) air quality would promote residents’ pro-environmental behavior. In other words, when people have close contact with air pollution, they are more willing to improve the ecological environment closely related to their own lives through their pro-environmental behavior. There was no significant difference between the effect of low air quality and high air quality on residents’ pro-environmental behavior intention at distant spatial distance. In other words, when people are far away from air pollution, they would lack the internal driving force to implement pro-environmental behavior. Second, in most cases, residents’ willingness to conduct private pro-environmental behavior is greater than that in the public domain, and the interactive effect of air quality and spatial distance on private pro-environmental behavior intention is greater. Third, environmental affection mediates the interactive effect of air quality and spatial distance on pro-environmental behavior intention. To be specific, within local spatial distance, environmental affection plays a mediating role between air quality and pro-environmental behavior intention. When people come into close contact with air pollution, they will be directly harmed by air pollution and will generate anxiety, guilt, disgust, and other environmental affections, thus driving their pro-environmental behavior. However, the mediating effect of environmental affection is non-significant at the distant spatial distance. When people are far away from air pollution, they tend to form the idea of “none of their own business” so that they may be less likely to generate environmental affection and to engage in pro-environmental behavior caused by the stimulation of environmental conditions.

### Theoretical Contributions

This study contributes to previous research through three main aspects.

#### Enrich the Research on Air Quality and Pro-environmental Behavior

Previous studies have put forward many influential theories in the field of pro-environmental behavior, such as TRA, TPB, NAM, and VBN, mainly focusing on the predictive power of individual personal variables on pro-environmental behavior (e.g., attitudes, norms, the sense of responsibility, values, knowledge, perceived behavioral control, etc.), and explored promoting pro-environmental behavior from the perspective of individual cognition and emotion as well as external economic and social conditions. However, the impact of natural environment conditions is rarely explored. Starting from the existing serious air pollution issue in China, this study discusses the effect of air quality within different spatial distance on the pro-environmental behavior of Chinese residents. Findings of this study verify the influence of external environmental conditions on the pro-environmental behavior of Chinese residents, expanding the research in the field of pro-environmental behavior. In addition, although air pollution has attracted the attention of scholars, the existing studies on air pollution mainly focus on the causes, hazards, prevention, and control methods of air pollution, paying little attention to its impact on residents’ behavior, especially pro-environmental behavior. In fact, air pollution resulted from human activities is a manifestation of environmental issues, which, in turn, may affect residents’ behavior. The conclusion of this study enriches the research in the field of air pollution and is helpful to better understand the complex relationship between air quality and pro-environmental behavior.

#### Provide an Important Perspective: Spatial Distance

This study explores the relationship of air quality and residents’ pro-environmental behavior from an important perspective: spatial distance. Although air pollution is a global environmental issue, its occurrence and influence scope are relatively local. Its influence on residents may also change with the difference of spatial distance. As a dimension of psychological distance, spatial distance can greatly influence individual’s perception of and response to external environment stimuli. Therefore, this study explores the relationship between air quality and pro-environmental behavior from the perspective of spatial distance. The conclusion of this study explains why many people are indifferent to environmental issues and unwilling to make behavioral changes from the perspective of spatial distance, which not only helps to verify the important role of spatial distance in effectively increasing residents’ pro-environmental behavior but also enriches the application of spatial distance in the field of pro-environmental behavior.

#### Present a Discussion on the Mediating Role of Environmental Affection

In this study, from the perspective of individual inner affection, environmental affection is introduced to explain the psychological mechanism of the interactive effect of air quality and spatial distance on Chinese residents’ pro-environmental behavior. Although existing studies have found that affection is one of the important antecedents of pro-environmental behavior, few studies have combined contextual factors with affection. This study combined psychological variables with natural contextual variables to explore the driving factors of Chinese residents’ pro-environmental behavior from multiple perspectives, enriching the research on the underlying mechanism of air quality to Chinese residents’ pro-environmental behavior.

### Implications

The findings of this study also provide practical implications for the policy-makers and business marketers committed to promoting pro-environmental behavior.

#### Make Rational Usage of Eco-Environmental Information

Policy-makers could release environmental information in a timely and accurate manner to ensure that the public has a full understanding of the state of the eco-environment. The findings of this study reveal that residents experiencing low air quality in close proximity would have a higher intention to conduct pro-environmental behavior. Due to the stimulation of air pollution, they would form a series of environmental affection toward environmental conditions and environment-related behavior and would thus be more willing to practice pro-environmental behavior. Therefore, the government could release environmental information such as the level of air quality to the public to let them have a clear perception of the environmental pollution issues, so as to stimulate their environmental affection and encourage them to actively engage in pro-environmental behavior driven by the affection.

#### Promote Pro-environmental Behavior From Both Public and Private Sectors

The results of this study show that compared with public pro-environmental behavior, people are more willing to engage in private pro-environmental behavior. The government could not only implement environmental protection into residents’ daily behavior but also encourage them to participate more in public pro-environmental activities, such as joining in environmental organizations and activities, making suggestions to relevant departments on environmental issues, and practicing the protection of trees and vegetation. It is important to give full play to the important role of social participation in environmental governance and to further expand the depth and breadth of public participation in environmental protection.

#### Develop Environmental Policies Tailored to Local Conditions

The government may formulate and implement appropriate environmental protection policies according to the conditions of the ecological environment in different regions. This study has found that residents have different affections for and reactions to environmental issues within different spatial distance. Residents in the regions with severe environmental pollution may more intuitively perceive the impact of the pollution. In such regions, the government could emphasize the effectiveness of residents’ pro-environmental behavior in improving environmental issues and encourage residents to improve the surrounding eco-environment through their own pro-environmental behavior, while for residents in the regions with good ecological environment, the government may focus on building public awareness of the environment, the overall view of the environment, and the sense of environmental responsibility. By means of publicity and education, the public could empathize with the environmental conditions of the country and even the whole world and then may be more willing to take practical actions.

#### Use Eco-Environmental Clues in Green Marketing Strategies

For enterprise marketers, environmental information clues can be appropriately used when formulating advertising or other publicity strategies to increase consumers’ intention to purchase green products. According to the conclusion of this study, low air quality within local area may promote private pro-environmental behavior. Green consumption is an important component of private pro-environmental behavior. Enterprises can reasonably use pollution-related advertising appeals to arouse consumers’ environmental affection, so as to make them more willing to purchase and use green products.

### Limitations and Future Research

This study still has the following limitations to be further discussed in the future. First, in this study, Changchun is taken as an example for experiments. Although foreign and domestic cities were selected in Experiment 2 and Experiment 3, respectively, to minimize the influence of control variables such as nationality and culture, there are still some other variables that may cause interference, such as customs and economic level. Therefore, the external validity of the results needs to be further tested in other regions. Second, the experiments of this study were conducted in the laboratory by means of image stimulation, and the intention to conduct pro-environmental behavior was measured by scale. In the future, field experiments can be conducted in the real environment to observe the actual behavior of the subjects, so as to expand the externality of the study. Third, the scale of pro-environmental behavior contains a variety of behaviors. Although this study compares the different intention of residents to private and public pro-environmental behavior, the preferences of residents for different kinds of pro-environmental behavior still need to be further discussed. Future research could further investigate how to measure the level of people’s intention toward different pro-environmental behavior. Finally, this study mainly focuses on the impact of air quality within different spatial distance on residents’ pro-environmental behavior. Whether other environmental issues such as soil pollution and water pollution would also influence the pro-environmental behavior of Chinese residents needs further discussion.

## Data Availability Statement

The raw data supporting the conclusions of this article will be made available by the authors, without undue reservation.

## Ethics Statement

The studies involving human participants were reviewed and approved by Department of Marketing, Business School, Jilin University. The patients/participants provided their written informed consent to participate in this study.

## Author Contributions

GS designed the experiments. GS and JD conducted the experiments and collected and analyzed the data. All authors wrote the first draft of the manuscript.

## Conflict of Interest

The authors declare that the research was conducted in the absence of any commercial or financial relationships that could be construed as a potential conflict of interest.
